# English and American Doctors

**Published:** 1893-07-15

**Authors:** 


### The Hospital

An Institutional Journal of

**Science, Medicine, Nursing, and Philanthropy.**

*For Hospitals, Asylums, Infirmaries, Dispensaries, Medical Practitioners, Students, Nurses, and the Charitable Public.*

Vol. XIV.—No. 355.] July 15, 1893.

### ENGLISH AND AMERICAN DOCTORS.

The new wine of modern medicine has burst the old wine-skins in America, and has furnished us with many curiosities, some examples, and a little wholesome inspiration. America is the continent of contrasts. On the same platform of public activity we see men more conservative than feudalism in its most unbending days, working side by side with irrepressibles and go-aheads, to whom tradition is anathema, and the checking of unbounded freedom the unpardonable sin. The strange thing is that, notwithstanding these extraordinary contrasts and contradictions, no cataclysms occur. It is true that every now and again some dignified conservative, as he witnesses outrageous departures from tradition, will "start ceiling high" in Carlylean phraseology, and have to be "borne home on shutters" But then, when he comes to think of it after a day or two, he finds even his own skin whole, and the great world thundering along on its mighty way as if nothing at all had happened. "I was perhaps a little unnecessarily precise," he tells himself; "possibly even pedantic and unscientific: I will use my thinking powers a little more and my prejudices a little less in future;" and there, in America, the matter ends.

In England the wine-skins are tougher, and the irrepressibles are fewer and less outrageous. But fermentation is fermentation even here. Development is a mighty energy which forces its resistless way in spite of a tradition which would die rather than yield. In England medicine must change her front; and she is changing it with some speed. In olden times the old-fashioned, half-educated, narrow-minded practitioner, enthroned in high places as physician to the Royal House, or surgeon to the Heir-apparent, laid down the law to a timid handful of similar practitioners, who called themselves Fellows of the Royal Colleges of Physicians and Surgeons, and they in turn assumed airs of authority and condescension towards the apothecaries who bled and blistered his Majesty's lieges in the streets of towns or in remote villages hidden away from the great lines of traffic. But that epoch is with us no more. It is true that there are here and there half cultured persons who have "scraped" through the examinations which lead to fellowships, and who, on the strength of such miraculous performances, affect to be men of weight and authority. But their claims are laughed to scorn by the thousands of University graduates who throng the ranks of family practice throughout the country. Dr. Fillgrave is as dead as dead can be, and all the elixirs which ever were invented can never bring him to resurrection. The medical practice of the future is the practice of the competent man everywhere, whether he be a fellow of the College of Physicians and live in London, or a member of the College of Surgeons and practice in the single street of Little Peddlington. The future of medicine in Great Britain is in the hands, not of a small clique of withered tradition-mongers in the metropolis, but of the thirty or forty thousand cultured, fearless, and independent-minded medical men, who in all sorts of places great and small practice the healing art.

The editors and conductors of about four hundred medical newspapers have recently met in congress in America. The bare announcement that there are four hundred medical newspapers in America almost takes the breadth away. How many have we in this country? Have we so many as four dozen? Have we, indeed, many more than four or five which a physiologist would declare to be living and in normal health? And yet the population of Great Britain is more than half the population of the whole of the United States. It is evident that a very different set of conditions obtains in America from that which exists in this country!

The most striking characteristics of modern medicine in America are courage and freedom. The American doctor almost makes our hair stand on end by his boldness. He is surely right if public opinion will stand by him. Timidity is the paralysis of intellect, the grave of progress. The most striking characteristic of modern medicine in this country is caution. This, too, is of great merit. But caution can be too cautious. We must move forward. Better is it to make crude experiments than to make no experiments at all. The misfortune in England is, that we assign experimentation to a handful and freedom to a class. The bold man claims his freedom, and we grant it to him because he has had the courage to take it. But the timid we forbid so much as to open his lips, except by permission. The whole profession and every man in it should claim his equal rights in everything that pertains to medicine. The man of real intellectual capacity desires above all things to see every member of his profession cultured, capable, courageous, and independent.

This note of independentmindedness—to Germanise a word—so characteristic of the medical profession in America, is the true and open secret of American progress. The day is dawning when a like independence will be universal in Great Britain. Oxford, Cambridge, and the other ancient universities are sending men of liberal culture and of highminded courage all over the country. These men will take no dictation from self-appointed censors and censurers. To what purpose have they devoted themselves to the study of the larger and more philosophical medicine, if the moment they enter upon the life of practice they are to cast themselves into the mental moulds of the ancients, who knew little or nothing about large and philosophical medicine? Freedom of thought, freedom of tongue and pen, freedom the largest and the most absolute, is the inheritance of every medical man as of every Englishman; and he is unworthy of the name of Englishman who at the bidding of college, clique, or medical editor casts away his noble heritage.

A little enthusiasm would put life into the somewhat rusty and creaking machine of English general medicine: a little enthusiasm and a little fearlessness. We are all too afraid of the critic, the terrible man with the omniscient pen who writes the reviews for the medical papers, or who poses as a *censor morum*. He will find out the weak places in our literary armour. He will proclaim to the medical world that we have presumed to write on a medical subject without having consulted the latest Timbuctoo authorities, or referred to the learned work of the distinguished Dr Kamschatka. How terrible is the reviewer to the timid author! How frightful is the censor of morals to the man who offends against medical etiquette by insisting upon following a consultant into the sick room instead of preceding him! The truly delicious part of the business is that the medical reviewer, poor creature, really imagines himself to be omniscient, and the censor of etiquette seriously regards himself as a casuist and an authority in ethics.

Good work well done, and the courage which rises spontaneously in the heart of the man who is conscious of such work, pays small heed to the irresponsible judgments of the mere newspaper scribbler. There should be a passion in the heart of every medical writer to see all his medical brethren men of courage and of generous independence of mind. Modern medicine is not a thing of mere unmeaning traditions and senseless etiquettes. It is a living science, a noble practical art. All its ethics and etiquettes which are worthy of the name come under the golden rule, "Whatsoever ye would that men should do unto you, do ye even so unto them." With this rule as the guiding star, medical men in England, like their brethren in America, may claim and use that bold and unshackled freedom which is the primest of all the conditions of progress and of true intellectual greatness.

